# Developing Bioprospecting Strategies for Bioplastics Through the Large-Scale Mining of Microbial Genomes

**DOI:** 10.3389/fmicb.2021.697309

**Published:** 2021-07-12

**Authors:** Paton Vuong, Daniel J. Lim, Daniel V. Murphy, Michael J. Wise, Andrew S. Whiteley, Parwinder Kaur

**Affiliations:** ^1^UWA School of Agriculture and Environment, The University of Western Australia, Perth, WA, Australia; ^2^Department of Physics and Astronomy, Curtin University, Perth, WA, Australia; ^3^School of Physics, Mathematics and Computing, The University of Western Australia, Perth, WA, Australia; ^4^Marshall Centre for Infectious Disease Research and Training, University of Western Australia, Perth, WA, Australia; ^5^Centre for Environment and Life Sciences, CSIRO, Floreat, WA, Australia

**Keywords:** bioplastic, bioprospecting, genome mining, PHA synthase, polyhydroxyalkanoates

## Abstract

The accumulation of petroleum-based plastic waste has become a major issue for the environment. A sustainable and biodegradable solution can be found in Polyhydroxyalkanoates (PHAs), a microbially produced biopolymer. An analysis of the global phylogenetic and ecological distribution of potential PHA producing bacteria and archaea was carried out by mining a global genome repository for PHA synthase (PhaC), a key enzyme involved in PHA biosynthesis. Bacteria from the phylum Actinobacteria were found to contain the PhaC Class II genotype which produces medium-chain length PHAs, a physiology until now only found within a few *Pseudomonas* species. Further, several PhaC genotypes were discovered within Thaumarchaeota, an archaeal phylum with poly-extremophiles and the ability to efficiently use CO_2_ as a carbon source, a significant ecological group which have thus far been little studied for PHA production. Bacterial and archaeal PhaC genotypes were also observed in high salinity and alkalinity conditions, as well as high-temperature geothermal ecosystems. These genome mining efforts uncovered previously unknown candidate taxa for biopolymer production, as well as microbes from environmental niches with properties that could potentially improve PHA production. This *in silico* study provides valuable insights into unique PHA producing candidates, supporting future bioprospecting efforts toward better targeted and relevant taxa to further enhance the diversity of exploitable PHA production systems.

## Introduction

Plastic waste pollution has become a significant issue, with plastic waste as of 2015 amounting to 6,300 million metric tons predicted to almost double to 12,000 million metric tons by 2050, with only 9% being recycled, 12% disposed of through incineration and 79% of which is sent to landfill or ending up in the natural environment (Geyer et al., 2017). The disposal processes for petroleum-based plastics are notoriously problematic—incineration is costly and produces toxic by-products and recycling is slow due to sorting required to accommodate the wide range of plastic formulations with certain plastic additives often limiting their use in recycling (Anjum et al., 2016). In addition, the production of plastics accounts for 4–8% of the global consumption of oil, with the projected use rising to 20% by 2050 (Narancic et al., 2020). The need for biodegradable and sustainable alternatives has, therefore, become critical with microbially produced polyhydroxyalkanoates (PHAs) showing significant promise as an economically viable replacement for petroleum-based plastic (Meereboer et al., 2020).

Polyhydroxyalkanoates (PHAs) are a group of microbially-made polyesters: 100% biodegradable, thermoplastic, insoluble in water, non-toxic and biocompatible. The major advantage for waste management is that PHA products are 100% biodegradable (compostable bioplastics) in the land and ocean, leaving no lasting waste management footprint. As such these biopolymers are well suited as a “green” alternative to petroleum-based plastics by being both biodegradable and non-toxic (Dietrich et al., 2017). Prokaryotic species that produce PHAs are broad and include many bacteria, such as *Alcaligenes latus, Ralstonia eutropha, Azotobacter beijerinckii, Bacillus megaterium* and *Pseudomonas oleovorans* (Bhuwal et al., 2013; Anjum et al., 2016), as well as several archaea from the family *Halobacteriaceae* (Han et al., 2010; Hermann-Krauss et al., 2013). PHAs are produced by microorganisms as a form of intracellular energy storage, within the microbial cytoplasm, and manifests when there is an excess supply of carbon but other essential nutrients, such as oxygen, nitrogen, and phosphorus are deficient (Laycock et al., 2014). The carbon-based “feedstocks” used in the development of efficient microbial PHA production are derived from sustainable and low-cost sources: agricultural waste (starch, lignocellulose and animal carcasses), molasses, whey, waste oils and glycerol from the production of biodiesel (Cui et al., 2016).

The types of PHAs produced by individual microorganisms are categorized by the number of carbon atoms within the monomer units that form the PHA polymer: short-chain length (SCL) PHAs consist of 3–5 carbon atoms, medium-chain length (MCL) PHAs with 6–14 carbon atoms and PHAs with > 14 carbon atoms are considered long-chain length (LCL) (Sagong et al., 2018). The mechanical properties of PHAs are influenced by the carbon length of the monomers, SCL-PHAs due to their crystalline structure, are generally stiff, brittle and have poor thermal stability, requiring modified processing approaches for an improved product (Wang et al., 2016). In comparison, MCL-PHAs have good thermos-elastomeric properties but are mainly limited to bacteria from the family *Pseudomonadaceae* (Oliveira et al., 2020). LCL-PHAs are rare and only produced by *Pseudomonas* strains (Meereboer et al., 2020) and have seen less interest within the area of bioplastic development (Muneer et al., 2020).

The enzyme responsible for the polymerization of monomeric substrates during PHA biosynthesis is PHA synthase (PhaC) which is organized into four classes depending on their primary protein structure, substrate specificity and subunit composition (Rehm, 2003). Class I contains a type of PhaC (∼60 kDa) which forms a homodimer; Class II has two synthases PhaC1 and PhaC2 (∼60 kDa each), in which only PhaC1 is physiologically active; Class III forms a heterodimer with a catalytic subunit PhaC (40–53 kDa) and a secondary subunit PhaE (20–40 kDa); and lastly, Class IV also forms a heterodimer with a catalytic subunit PhaC (41.5 kDa) and a secondary subunit PhaR (22 kDa) (Mezzolla et al., 2018). Class I and IV PhaCs favor SCL monomers, with carbon chain lengths C3-C5 whilst Class II PhaC prefer MCL monomers, composed of carbon chain lengths C6-C14 (Chek et al., 2017). Class III PhaCs also favor C3-C5 SCL monomers but can also utilize substrates with C6-C8 carbon chain lengths (Rehm, 2003; Ray and Kalia, 2017).

More than 150 unique monomers have been discovered within PHA polymer samples since the discovery of PHA (Agnew and Pfleger, 2013). PHA polymer properties meet the design needs of nearly all synthetic plastics providing considerable industrial product design and manufacturing flexibility. As an example, PHAs have physical properties similar to synthetic polyethylene and polypropylene (polyolefins) which are used extensively in single-plastic use products which are at higher risk of becoming waste. The composition of the monomers is tied to the substrate specificity of the individual PhaC enzyme, and PHAs with novel monomer compositions are constantly being discovered from bacteria in various environments (Sharma et al., 2017). PhaCs with broad substrate specificity are desirable for two major reasons. PHA with co-polymer composition have been observed to possess better physical properties compared to homopolymer SCL-PHAs (Chek et al., 2017; Ray and Kalia, 2017). In addition, they can more readily utilize a wider range of carbon substrates which could potentially include inexpensive plant or animal waste products (Nielsen et al., 2017; Surendran et al., 2020).

Microbial bioprospecting of large genome collections for the presence of PhaCs could be a viable approach to discover new substrate specificities and monomer compositions that could improve current PHA production systems. Genome mining is a method of *in silico* bioprospecting where collections of genome sequences are searched for putative enzymes involved in the biosynthesis of secondary metabolites (Ziemert et al., 2016). The Integrated Microbial Genomes and Microbiomes system (IMG/M) is an online platform that allows users access to a global repository of genome and metagenome datasets (Chen et al., 2021). The Genomes OnLine Database (GOLD), provided through IMG/M, is a manually curated database with a metadata reporting system that allows users to easily tabulate and browse the associated metadata assigned to each submitted genome (Mukherjee et al., 2021). Exploring the global distribution of microorganisms along with access to large scale ecological data could give valuable insight toward taxa that are less studied, as well as environmental microbes that exist in extreme or unusual ecosystems, for their potential in improving current PHA production methods.

## Materials and Methods

### Preparation of Bacterial and Archaeal Data

A set of 16,576 bacteria (accessed 14th Dec 2020) and 1932 archaea (accessed 20th Feb 2021) genome sequences along with their associated taxonomic and ecosystem metadata were obtained from the IMG/M^1^ warehouse to create the experimental dataset (Supplementary Data 1, 2). Due to the large volume of bacterial sequences available, only the JGI sequenced bacteria genomes were obtained for this study, whereas all available archaeal genomes (both JGI and externally sequenced) were downloaded. PhaC protein sequences used to query the genome sequences were sourced from UniprotKB^2^ database using the search term “gene:phac” and included both reviewed (Swiss-Prot) and unreviewed (TrEMBL) results (The UniProt C, 2021). The bacterial PhaC protein sequences were filtered using the term “taxonomy:bacteria” with a resulting 4,038 sequences (accessed 21st Jan 2021) and archaeal sequences with “taxonomy:archaea” resulting in 178 sequences (accessed 20th Feb 2021). Available protein sequences retrieved from UniprotKB were limited to Class I, II, and III PhaCs for bacterial sequences and Class I and III for archaeal sequences. Bash^3^ and MATLAB^4^ were used to conduct the sorting and categorization of the large-scale data. The scripts are available in our GitHub^5^ repository.

### PhaC Reference Sequence Preparation and Curation

The sequences were first manually curated through visual inspection of the metadata. The following categories of sequences were removed through inspection of the protein name: (1) Names determined to not be PhaCs, (2) Names with ambiguous function, (3) Putative PhaCs and (4) Fragment PhaCs. PhaC sequences with amino acid lengths < 328 in bacteria and < 100 in archaea were also filtered out as “fragments.” Duplicate protein sequences were removed. Subsets of the PhaC sequences created according to the class listed in the protein name field from the respective metadata entry. Where the protein name entry did not identify the class, the TIGRFAM metadata entry was used to assign the PhaC class.

BLAST + v2.10.1 was used to classify the remaining unclassified PhaCs (Camacho et al., 2009). The combined subset of known PhaC (Class I, II, and III for bacterial PhaC and Class I and III for archaeal PhaCs), were aligned *via* BlastP with e-value = 1 × 10^–10^ against the unclassified PhaC sequences of the respective taxonomic domain. The unclassified sequences were then classified according to the class of the query sequence with the lowest e-value. In cases where multiple hits achieved the lowest e-value, the query sequence with the highest bitscore was used for classification. The final curated protein sequence dataset used for the prediction of PhaCs in the genome datasets were 2,799 bacterial PhaCs (Class I = 1,799; Class II = 604; Class III = 375; Unknown = 1) and 140 archaeal PhaCs (Class I = 2; Class III = 137; Unknown = 1). The final curated bacteria and archaea query PhaC protein sequences and associated metadata are available in Supplementary Data 3, 4, respectively.

### PhaC Distribution in the Genome Datasets

The PhaC protein sequences were aligned against their respective domain genome sets (Supplementary Data 1, 2) using TblastN with e-value = 1 × 10^–10^. A single best hit was chosen from each genome as the representative PhaC protein sequence for use in downstream phylogenetic comparisons by selecting the lowest e-value result. In cases where multiple hits achieved the lowest e-value, the hit with the highest bitscore was used as the representative predicted PhaC sequence. The UniprotKB ID of the matching query, as well as PhaC Class, were appended onto the taxonomic and ecosystem metadata of IMG genomes for identified PhaC genotypes and can be found in Supplementary Data 5, 6 for bacteria and archaea, respectively. Multiple sequence alignment was performed using both the BLAST predicted PhaC sequences within genomes and the curated Uniprot reference sequences *via* Kalign 3, with default settings (Lassmann, 2020). The alignment was performed for all PhaC protein sequences within their respective domains to visualize the distribution of PhaC classes. Due to the large number of sequences, FastTree v2.1.10 (Price et al., 2010) was used to create maximum-likelihood phylogenetic trees using the WAG amino acid substitution model, implemented through the Galaxy^6^ online platform (Afgan et al., 2016). Phylogenetic trees were visualized using the Interactive Tree of Life^7^ (iTOL) online tool (Letunic and Bork, 2019).

## Results

### Phylogenetic Distribution of Bacterial PhaCs Genotypes

**FIGURE 1:**
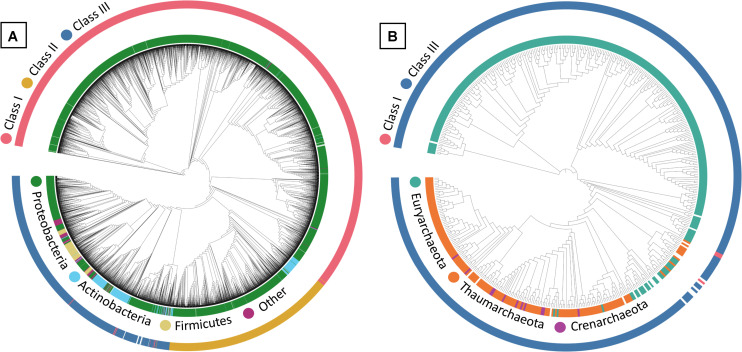
Maximum-likelihood tree of **(A)** bacterial and **(B)** archaeal PHA Synthase protein sequences. Sequences include both UniprotKB query sequences and BLAST identified sequences from IMG genomes. Uncolored strip sections indicate unclassified data. Created using Interactive Tree of Life (iTOL).

Within the maximum-likelihood tree, the bacterial PhaC protein sequences appeared to form generally distinct groupings within their respective classes in the phylogenetic tree, with Class III PhaCs showing relatively high variation in phyla compared to those seen in Class I and II PhaCs (Figure 1A). PhaCs of unknown class were included to determine if their classification could be inferred *via* phylogenetic tree reconstruction. From the phylogenetic tree, the unknown class sequences appear to fall within the majority Class III grouping on the tree. However, the placements appear to be close to a variable region where different PhaC classes are dispersed and as such tentatively remained unclassified and were omitted from further analysis.

From the initial bacterial genome dataset PhaCs were identified in 24.8% (*n* = 4,119) of the genomes. From this subset of genomes with PhaC genotypes, 2339 Class I, 529 Class II and 1,229 Class III PhaCs were identified according to the class of the matching query sequence used in the BLAST search (Supplementary Data 5). Class I PhaCs from the genome dataset were identified predominantly in Proteobacteria comprising 98.2% (*n* = 2,298) of the Class I containing genomes with the remaining 1.8% comprising of Actinobacteria (*n* = 26), Bacteroidetes (*n* = 12), Candidatus Dadabacteria (*n* = 1), Chloroflexi (*n* = 1) and Spirochetes (*n* = 1). Within Class II PhaC identified genomes, Proteobacteria were also dominant appearing in 81.5% (*n* = 431) of the genomes with the remaining 18.5% from Actinobacteria (*n* = 97) and Bacteroidetes (*n* = 1). Class III PhaCs in comparison were identified in a much larger variety of phyla although Proteobacteria still remaining the largest contributor at 51.8% (*n* = 637), followed by Actinobacteria 27.7% (*n* = 341), Firmicutes 12% (*n* = 148), with the remainder consisting of established phyla 5.4%; Acidobacteria (*n* = 5), Bacteroidetes (*n* = 13), Chloroflexi (*n* = 9), Cyanobacteria (*n* = 17), Elusimicrobia (*n* = 3), Gemmatimonadetes (*n* = 2), Nitrospirae (*n* = 3), Planctomycetes (*n* = 3), Spirochetes (*n* = 9), Verrucomicrobia (*n* = 2) and from candidatus or unknown phyla 3.1%; candidate division NC10 (*n* = 1), Candidatus Blackallbacteria (*n* = 3), Candidatus Dadabacteria (*n* = 1), Candidatus Falkowbacteria (*n* = 3), Candidatus Kaiserbacteria (*n* = 2), Candidatus Melainabacteria (*n* = 3), Candidatus Moranbacteria (*n* = 1), Candidatus Riflebacteria (*n* = 1), Candidatus Rokubacteria (*n* = 15), Candidatus Wallbacteria (*n* = 1), and unclassified (*n* = 6).

### Environmental Distribution of Bacterial PhaCs Genotypes

**FIGURE 2:**
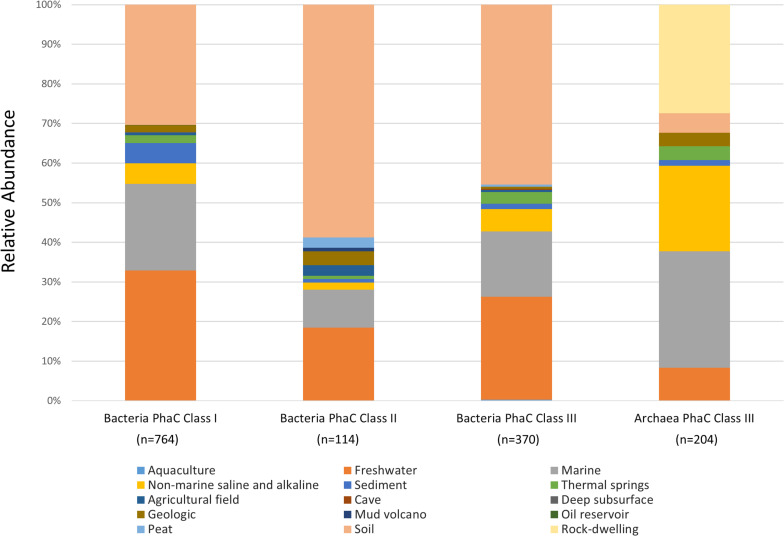
Environmental distribution of bacteria and archaea PHA Synthase genotypes. The relative abundance of IMG Genomes with classified GOLD “Aquatic” or “Terrestrial” ecosystem categories.

To determine the distribution of bacteria PhaC genotypes in relevant environments, genomes were used if marked as “environmental,” under the GOLD ecosystem category, or “aquatic” or “terrestrial” under the GOLD ecosystem category, in the metadata contained in Supplementary Data 5. Entries that were “unclassified” were not pertinent regarding environmental information and were omitted. From the identified PhaC genotypes, environmental genomes were observed containing 765 Class I, 114 Class II and 370 Class III PhaCs (Figure 2).

Class I PhaC genotypes were most abundant in diverse environments with 32.8% (*n* = 251) present in “Freshwater,” 30.4% (*n* = 232) in “Soil” and 21.8% (*n* = 167) from “Marine” ecosystem types. Class I were also present in other aquatic ecosystem types consisting of; 5.2% (*n* = 40) “Non-marine saline and alkaline,” 5.1% (*n* = 39) “Sediment” and 2% (*n* = 15) “Thermal springs.” The remaining 2.7% appeared in small amounts from terrestrial environments; (*n* = 5) “Agricultural field,” (*n* = 1) “Deep subsurface” and (*n* = 1) “Oil reservoir.” Within Class I bacterial genotypes, the presence of Proteobacteria were prevalent across all observed ecosystem types, with trace representations of Actinobacteria in “Soil” and “Thermal springs,” Bacteroides in “Freshwater” and “Soil,” Chloroflexi in “Marine,” Spirochetes in “Freshwater” and Candidatus Dadabacteria in “Soil” (Supplementary Table 1).

Over half of Class II PhaC genotypes were found in “Soil,” with 58.8% (*n* = 67) representation with the second and third largest percentage consisting of diverse aquatic environments, 18.4% (*n* = 21) “Freshwater” and 9.6% (*n* = 11) “Marine” ecosystem types. The rest of the Class II genotypes were found in the remaining 7% terrestrial environments; (*n* = 4) “Geologic,” (*n* = 3) “Agricultural field,” (*n* = 3) “Peat” and (*n* = 1) “Mud volcano” as well as 3.6% aquatic environments; (*n* = 2) “Non-marine saline and alkaline,” (*n* = 1) “Sediment” and (*n* = 1) “Thermal springs.” From the Class II genotypes, Proteobacteria was present across almost all observed ecosystem types, with Actinobacteria being the only other phylum present, found mostly within “Soil” and in trace amount across “Freshwater,” “Marine” and “Geologic” and the sole representative within “Mud volcano” (Supplementary Table 2).

Class III PhaC genotypes followed the trend of having the highest percentage of abundance in diverse terrestrial and aquatic environments with 45.4% (*n* = 168) in “Soil,” 25.9% (*n* = 96) “Freshwater” and 16.6% (*n* = 61) from “Marine” ecosystem types. Class III genotypes were present in other aquatic environments with relative abundances of; 5.7% (*n* = 21) “Non-marine saline and alkaline,” 3% (*n* = 11) “Thermal springs,” 1.4% (*n* = 5) “Sediment” and 0.3% (*n* = 1) “Aquaculture” with the remaining 1.8% from terrestrial ecosystem types; (*n* = 2) “Agricultural field,” (*n* = 2) “Geologic” and (*n* = 1) “Cave.” Compared to Class I and II, Class III genotypes were highly diverse across the observed ecosystem types (Supplementary Table 3). The ecosystem types containing Class III genotypes of note were “Freshwater” and “Soil,” with the highest levels of diversity across all PhaC classes and were observed to contain representatives of several Candidatus phyla.

Environmental bacterial PhaC genotypes also investigated to determine whether they were recovered from unusual or niche settings (Table 1). Within each class a total of 8.6% (*n* = 66) Class I, 5.4% (*n* = 6) Class II and 10.8% (*n* = 40) Class III PhaC genotypes were found in ecosystems with extreme physicochemical conditions. The most common niche environments present across all bacterial PhaC genotypes were high salinity and alkalinity conditions found in “Non-marine saline and alkaline” ecosystem types and the unique high-temperature geothermal environments found in “Thermal springs” and ‘Mud volcano” ecosystem types as well as “Hydrothermal vents” found in “Marine” ecosystem types. Bacteria PhaC genotypes were also found in acidic, desert, saline or permafrost environments, as well as ecosystems with petroleum contamination: “Oil reservoir” and “Creosote-contaminated soil.” The distribution of phyla across these niche ecosystem settings for Class I, II and III bacterial genotypes can be found in Supplementary Tables 1–3, respectively.

**TABLE 1:** Environmental PhaC genotypes found in extreme physicochemical conditions.

Ecosystem Type	Extreme ecosystem subtype or habitat descriptors	Bacteria PhaC			Archaea PhaC
		Class I	Class II	Class III	Class III
Marine	Deep oceanic, basalt-hosted subsurface hydrothermal fluid, Hydrothermal vents	3	2	4	3
	Deep-sea				3
	Creosote-contaminated soil			1	
Non-marine saline and alkaline		40	2	21	44
Thermal springs		15	1	11	7
Geologic	Acid Mine	2			
	Saline water, Salt, and Salt mine	4			3
Oil reservoir		1			
Mud volcano			1		
Rock-dwelling	Halite pinnacle				55
Soil	Creosote-contaminated soil			1	
	Desert soil	1		1	
	Permafrost sediment			1	
	Saline				3
Subtotal (Relative abundance)		66	6	40	118
		(8.6%)	(5.3%)	(10.8%)	(57.8%)

*Descriptors are taken from GOLD metadata under the ecosystem subtype or habitat headings. Ecosystems with no descriptors inherently contain extreme conditions.*

### Phylogenetic Distribution of Archaeal PhaCs Genotypes

In the maximum-likelihood tree, most of the archaeal PhaC protein sequences belonged to Class III PhaCs, with the few Class I and unclassified Class PhaCs sequences appeared to group closely together apart from the Class III PhaCs (Figure 1B). Due to the ambiguity of the unknown class PhaCs, they were omitted from further downstream analysis. Within the Class III PhaCs, a distinct grouping can be observed from Class III PhaCs belonging to Euryarchaeota compared to the Class III PhaCs found in Crenarchaeota and Thaumarchaeota. From the initial dataset of archaeal genomes, there were PhaCs predicted in 17.5% (*n* = 338) of the samples (Supplementary Data 6). The sole genome containing Class I (*n* = 1) belonged to the phylum Euryarchaeota. Class III was the dominant PhaC genotype appearing within the archaea genomes with 65.4% belonging to Euryarchaeota (*n* = 217), 30.1% from Thaumarchaeota (*n* = 100), 3% from Crenarchaeota (*n* = 10) and the remaining 1.5% unclassified (*n* = 5).

### Environmental Distribution of Archaeal PhaCs

Within the archaeal PhaC identified genomes, only Class III PhaC genotypes had representative samples from environmental settings according to the GOLD metadata listed in Supplementary Data 6 (Figure 2). The highest abundance environments that archaea Class III genotypes were present in were 29.4% (*n* = 60) “Marine,” 27.4% (*n* = 56) “Rock-dwelling” and 21.6% (*n* = 44) “Non-marine saline and alkaline” ecosystem types. Contrary to the bacteria PhaCs, archaea Class III PhaCs genotypes were less represented in the diverse ecosystem with abundances of 8.3% (*n* = 17) in “Freshwater” and 4.9% (*n* = 10) from “Soil” with the remaining found in 3.4% (*n* = 7) “Thermal springs,” 3.4% (*n* = 7) “Geologic” and 1.6% (*n* = 3) from “Sediment” ecosystem types. Euryarchaeota were present across all observed ecosystem types, with Thaumarchaeota present in “Freshwater,” “Marine,” “Thermal springs” and “Soil,” Crenarchaeota present solely in “Marine,” and unclassified archaeal phyla within “Marine” and “None-marine saline and alkaline” (Supplementary Table 4).

Over half of the archaea Class III genotypes were found in extreme settings with 57.8% of all archaea PhaC environmental genomes from ecosystems with extreme physicochemical conditions (Table 1). The two settings that formed the majority of extreme ecosystems were the saline “Halite pinnacle” found in almost all the “Rock-dwelling” ecosystem types as well as the high salinity and alkalinity “Non-marine saline and alkaline” ecosystem types. Aside from other saline environments, archaea PhaC genotypes were also found in the “Marine” ecosystems within “deep sea,” and “Hydrothermal vents” habitats as well as from “Thermal springs” ecosystems. The distribution of archaeal phyla within the observed niche settings can be found in Supplementary Table 4.

## Discussion

The genome mining effort has provided an encompassing view of the phylogenetic and ecological distribution of bacterial and archaeal PhaC genotypes. This *in silico* microbial bioprospecting method presents a broad categorization method that inexpensively searches publically available data for PHA producing candidates across multiple bacterial and archaeal phyla. Through this approach, we were able to visualize the presence of different PhaC classes amongst various phyla and ecosystems. The resulting data provided insights on the general distribution of PhaC genotypes including ecological “hot spots,” as well as presenting microbial candidates that could improve current PHA production systems by utilizing cheaper feedstock or providing more efficient operating conditions.

PhaC genotypes appeared in a broad number of bacterial and archaeal genomes, consisting of 24.8 and 17.5%, respectively of the genome datasets, suggesting that PHAs are a relatively conserved form of carbon energy storage within bacteria (Serafim et al., 2018) and archaea (Wang et al., 2019b). This is also supported by the distribution of Class III PhaCs in bacteria, which the study identified across 23 phyla, roughly one-fifth of the 111 recognized bacterial phyla by the Genome Taxonomy Database^8^ through the standardization of bacterial taxonomy (Parks et al., 2018). Class III PhaCs have more variation in PHA chain lengths compared to other SCL-PHA producing classes, which may lead to a great potential of unique monomer subunits (Ray and Kalia, 2017). Combined with the large scale of observed diversity, Class III PhaCs may harbor a wider range of substrate specificities that could potentially produce novel monomeric compositions which could be the target for protein engineering for improved PHA properties and production (Jia et al., 2016). The findings suggest that PhaCs, in particular Class III genotypes, are more prevalent and diverse in composition within both bacteria and archaea domains through our *in silico* observations.

The dominance of Proteobacteria across Class I and II and roughly half of Class III PhaC genotypes suggest that PHA production is highly conserved within the phylum. The prevalence of Proteobacteria as PHA producers was similarly observed in a study investigating PHA accumulating organisms in a mixed microbial culture, with Proteobacteria accounting for nearly 88% of the microbial community when the PHA content was at its peak level (Sruamsiri et al., 2020). Proteobacteria have been observed to have highly diversified genomes that enables metabolic adaptability in response to varying environmental conditions (Zhou et al., 2020). This indicates that Proteobacteria contain innate physiology which more readily responds to changes to external stressors, with PHA production as one of the responses to fluctuations in available nutrient levels. Proteobacteria genotypes contain Class I, II and III PhaCs, which demonstrates a wide variation in substrate specificity, likely owing to the adaptive nature of the diversified genomes. However, this does not explain the almost exclusivity of Class I and II PhaCs to Proteobacteria and the relatively high taxonomic diversity seen in Class III PhaCs. Within the PHA gene cluster, *phaC* has been identified as the hub gene and plays a major role in cluster formation in prokaryotes (Kutralam-Muniasamy et al., 2017). Further study is required to uncover why Class III PhaC genotypes presumably undergoes more horizontal transfer compared to Class I and II and why Class III cluster appeared to be favored in a higher variety of taxa.

A potential MCL-PHA producing alternative the study discovered is bacteria from the phylum Actinobacteria, which was amongst the Class II PhaC genotypes that were identified in our studies. Class II PhaCs are of particular interest as they are the only PhaC class capable of producing MCL-PHAs and bacteria that generate MCL-PHAs are mainly *Pseudomonas* which consists of almost all of the Proteobacteria PhaC II genotypes that were found in this study (Mozejko-Ciesielska et al., 2019). MCL-PHAs have not had widespread production due to requiring expensive carbon sources needed for *Pseudomonas* PHA production, meaning cheaper compatible feedstocks were needed to be found (Tanikkul et al., 2020), or recombinant strains are required to utilize inexpensive industry by-products as substrates (Löwe et al., 2017). Actinobacteria is widely recognized within sustainable industries for its ability to breakdown plant biomass including the use of agricultural and forestry plant waste as a carbon source (Lewin et al., 2016; Bettache et al., 2018). Since raw materials can account for 40–48% of production costs, bioprospecting for Actinobacteria Class II PhaC genotypes could potentially kickstart industrial levels of MCL-PHAs through the use of inexpensive feedstock (Schmidt et al., 2016).

Roughly two-thirds of the archaea PhaC genotypes found in this study were from the phylum Euryarchaeota, of which most of the constituents were class Halobacteria. The abundance of Halobacteria PhaC genotypes and the favourability of extremophiles to work in broad conditions is reflected in the almost exclusive focus on haloarchaea in the study of archaea PHA producers (Han et al., 2010; Legat et al., 2010; Hermann-Krauss et al., 2013). Our search identified several PhaC genotypes within the archaeal phylum Thaumarchaeota within aquatic ecosystems, and to date, no extensive studies have been performed on the viability of PHA production within this phylum. Thaumarchaeota are found across many ecosystems, including extreme environments such as acidic and alkaline soils as well as in trenches within the hadal zone of the ocean (Wang et al., 2019a; Zhong et al., 2020). The CO_2_ fixation pathway is highly efficient even in nutrient-limited conditions within Thaumarchaeota, meaning it could potentially utilize CO_2_ as a cheap and sustainable feedstock for PHA production (Könneke et al., 2014; Reji and Francis, 2020). With a broad range of operation and efficient utilization of an inexpensive carbon source, Thaumarchaeota could be considered a viable PHA production candidate.

The environment with extreme conditions that PhaC genotypes from both bacteria and archaea were observed to appear most in was the “non-marine saline and alkaline” ecosystem type. Soda lakes are the prime example of this ecosystem type being saline, high pH environments with high amounts of available carbon in the form of carbonates and bicarbonates, often with limited levels of nitrogen compounds—conditions that favor the production of PHAs (Ogato et al., 2015). Despite appearing as inhospitable environments, they are host to a diverse array of microbial life, which includes multiple bacterial phyla and the species from the Euryarchaeota phylum of archaea (Sorokin et al., 2014). With the right conditions for PHA production as well as positive genotyping of PhaCs from both domains from our study, high salinity, high pH environments such as soda lakes could be a potential trove of PHA producing microbes for future microbial bioprospecting ventures.

Another extreme physicochemical environment of note from our findings into the ecological distribution of PhaC genotypes were geothermal ecosystems such as hydrothermal vents or hot springs. Hydrothermal vents are areas of the ocean floor where seawater percolates through cracks in the crust and are superheated by rocks in contact with magma (Dick, 2019). The areas, around which hydrothermal fluid circulate, are rich in carbon and are dominated by microbial thermophilic methanogens (Minic and Thongbam, 2011; Ver Eecke et al., 2012). Hot springs are similar in setting, except the water source comes from groundwater and is also rich in microbial life (Urschel et al., 2015; Narsing Rao et al., 2021). From observation of environmental PhaC genotypes from niche environments, it seems that PHA producers can be readily found in extremophiles containing extreme physicochemical conditions, so long as there is an abundant carbon source.

Over half of the archaea PhaC genotypes were found in extreme environments, which may indicate that taxa that commonly contain extremophiles may have developed physiological strategies utilizing PHA production. Within extreme environments, PHA production in microbial extremophiles acts not only as a carbon energy reserve but may also provide a protective role by maintaining cellular and membrane integrity against extreme physiochemical conditions (Obulisamy and Mehariya, 2021). PHA has been shown to function alongside extracellular polysaccharides (EPS) in microbial extremophiles to protect cellular function and structure from physiochemical stress (López-Ortega et al., 2021). Studies have found that in *Haloferax mediterranei*, a species of halophilic archaea, changing stressors such as increasing salinity (Cui et al., 2017a) or making nitrogen levels deficient (Cui et al., 2017b) can promote the accumulation of PHA over that of EPS. These findings provide insight into how PHA performs a critical role in microbial survival against external stress factors, as well as strategies on how to apply these stress factors to improve accumulation rate during PHA production.

PhaC genotypes discovered from these extreme environments could prove beneficial for cost reduction in industrial PHA production. Factors that contribute to the high cost of PHA production include maintaining a closed, contamination-free environment for fermentation/growth, expensive feedstock and difficult PHA extraction processes (Chen et al., 2020). Utilizing halophile PHA producers conveys several advantages in this regard: a high salinity environment can undergo continuous fermentation in an open unsterile environment which reduces operating costs, they can use various inexpensive feedstocks to produce PHAs and due to high intracellular osmotic pressure of halophiles, they can be lysed using tap/municipal water for easy PHA extraction (Mitra et al., 2020). Thermophiles can similarly reduce contamination risks by using elevated temperatures during fermentation (Xiao et al., 2015) and have been investigated for use in sustainable industries for their thermostable bioproducts (Debnath et al., 2019). By exploiting the extreme conditions that certain microbes have adapted to, we may be able to develop processes that can lead to more efficient and cost-effective PHA production techniques.

Studies screening for PHA synthase sequences recovered from niche and under-represented environments has yielded success in novel insights toward diversifying PHA production methods. Genomes from *Janthinobacterium* sp. isolates recovered from Antarctica were found to contain hitherto uncharacterized putative PHA synthase genes that were phylogenetically divergent from the known PhaC classes and tentatively labeled as “Class V” PhaCs (Tan et al., 2020). The first occurrence of PHA producers from hypersaline microbial mats were discovered in salt concentration ponds located in Mexico through PCR amplification of *phaC* genes (Martínez-Gutiérrez et al., 2018). A study which mined PHA synthases from mangrove soil metagenomes in Malaysia uncovered a novel synthase from unculturable bacteria that was able to utilize a wide range of substrates by incorporating six types of PHA monomers, demonstrating that PHA sequences procured from *in silico* based approaches are effective for diversifying PHA production methods (Foong et al., 2018). The aforementioned studies demonstrate the versatility of *in silico* screening methods for directing future strategies toward selecting promising candidates and novel synthases for PHA production strategies.

The creation of a categorized genomic list of potential PHA producing microbes presents a valuable resource that directs researchers to explicit candidates, as well as access to potential metabolic pathways of interest. A similar bioprospecting strategy was utilized successfully by Bustamante et al. (2019) using *in silico* screening methods to screen for putative PHA and β-galactosidase genes in a select number of microorganisms from literature to find PHA producers that could hydrolyze lactose for utilizing excess whey produced from the dairy industry as a cheap feedstock alternative. In comparison, our study has presented a broader scope by using genomes from large-scale data repositories in the public domain, allowing researchers access to a wider array of candidates which can be sorted by PhaC class, taxonomy and ecological information. Through this resource, users can filter for candidates of interest for and can locate the resulting genome for further analysis of metabolic activity relevant to the production method being investigated.

This study is a proof of concept that contextual information generated from large-scale genome mining is highly valuable for use in developing bioprospecting strategies with regards to both ecological and taxonomic data. This approach can be applied to any target functional protein or gene of interest to create a shortlist of microbial candidates and their potential ecosystems for further in-depth analysis or experimentation. The effectiveness of this approach is dependent on not only the volume of the available sequence data, but also on prudent reporting and ready access to metadata. Accompanying information such as physiochemical conditions, geolocation and other ecologically contextual data are often missing from sequence data recovered from environments, which is critical for the eventual recovery of isolates (Gutleben et al., 2018). To address this issue, reporting protocols have been proposed such as the Minimum Information about any (x) Sequence (MIxS) by the Genome Standards Consortium^9^ and the GOLD metadata system by utilized by IMG/M being examples of creating standards for robust metadata reporting. The continual growth of online sequence repositories within the public domain and improvement of standards for metadata reporting presents a massive pool of data freely available to exploit. The mining of large-scale data provides a broader view into patterns in environmental or taxonomic distribution of biosynthetic products, which only improves over time as more data is constantly accumulated from studies conducted across the globe.

## Conclusion

Genome mining allows for the large-scale discovery of potential microbial PHA producers through *in silico* identification of PhaC genotypes. Through the categorization of genotypes *via* phylogenetic and ecological information, we can find insight through the discovery of less-studied taxa as well as microbes from environmental niches with potential properties that can improve on current PHA production techniques. Although this approach is no substitute for protein expression assays, it presents an overview of the trends inherent in PHA producing microbes. The information gained from this study could be utilized for directing bioprospecting ventures for more effective discovery of potential PHA producing microbes.

## Data Availability Statement

The original contributions presented in the study are included in the article/Supplementary Material, further inquiries can be directed to the corresponding author/s.

## Author Contributions

PV and DL performed the research under the guidance of PK, DM, MW, and AW. PV wrote the manuscript with contributions from DL, PK, DM, MW, and AW. All authors read the manuscript and approved the content.

## Conflict of Interest

The authors declare that the research was conducted in the absence of any commercial or financial relationships that could be construed as a potential conflict of interest.

## Footnotes

1 https://img.jgi.doe.gov/cgi-bin/m/main.cgi

2 https://www.uniprot.org/

3 https://www.gnu.org/software/bash/

4 https://www.mathworks.com

5 https://github.com/pvuonguwa/phac_dist

6 https://usegalaxy.org.au

7 https://itol.embl.de

8 https://gtdb.ecogenomic.org/

9 https://gensc.org/mixs/

## Supplementary Material

Supplementary Table 1 Bacteria class I PhaC genotype environmental distribution.

Supplementary Table 2 Bacteria class II PhaC genotype environmental distribution.

Supplementary Table 3 Bacteria class III PhaC genotype environmental distribution.

Supplementary Table 4 Archaea class III PhaC genotype environmental distribution.

Supplementary Data 1 List of Bacterial Genomes retrieved from IMG/M with ID and accompanying metadata.

Supplementary Data 2 List of Archaeal Genomes retrieved from IMG/M with ID and accompanying metadata.

Supplementary Data 3 Curated Uniprot bacteria PhaC protein sequences. Contains protein sequences along with taxonomic metadata and Uniprot ID. Sequences that did not contain PhaC class information from Uniprot were classified *via* BLAST against PhaCs with known class.

Supplementary Data 4 Curated Uniprot archaea PhaC protein sequences. Contains protein sequences along with taxonomic metadata and Uniprot ID. Sequences that did not contain PhaC class information from Uniprot were classified *via* BLAST against PhaCs with known class.

Supplementary Data 5 Dataset containing IMG/M bacterial genomes with PhaC genotypes predicted from BLAST. Each IMG genome contains taxonomic and ecological GOLD metadata as well as the corresponding Uniprot ID from BLAST best hit.

Supplementary Data 6 Dataset containing IMG/M archaea genomes with PhaC genotypes predicted from BLAST. Each IMG genome contains taxonomic and ecological GOLD metadata as well as the corresponding Uniprot ID from BLAST best hit.
