# Supplementary material for: Developing Bioprospecting Strategies for Bioplastics Through the Large-Scale Mining of Microbial Genomes
Source: Front Microbiol. 2021 Jul 12;12:697309. doi: 10.3389/fmicb.2021.697309 (PMC8312272; doi:10.3389/fmicb.2021.697309)
Supplement: Supplementary Table 1 — Bacteria class I PhaC genotype environmental distribution. [file Table_1.docx]

**Supplementary Table 1** | Bacteria class I PhaC genotype environmental distribution.

| Environment | Type | Phylum | Found in extreme conditions? | | |
| --- | --- | --- | --- | --- | --- |
|  |  |  | No | Yes | Subtype or habitat descriptors |
| Aquatic | Freshwater | Bacteroidetes | 6 |  |  |
|  |  | Proteobacteria | 244 |  |  |
|  |  | Spirochaetes | 1 |  |  |
|  | Marine | Chloroflexi | 1 |  |  |
|  |  | Proteobacteria | 163 |  |  |
|  |  |  |  | 3 | Deep oceanic, basalt-hosted subsurface hydrothermal fluid, Hydrothermal vents |
|  | Non-marine saline and alkaline | Proteobacteria |  | 40 |  |
|  | Sediment | Proteobacteria | 39 |  |  |
|  | Thermal springs | Actinobacteria |  | 1 |  |
|  |  | Proteobacteria |  | 14 |  |
| Terrestrial | Agricultural field | Proteobacteria | 5 |  |  |
|  | Deep subsurface | Proteobacteria | 1 |  |  |
|  | Geologic | Proteobacteria | 9 |  |  |
|  |  |  |  | 2 | Acid mine |
|  |  |  |  | 4 | Salt mine |
|  | Oil reservoir | Proteobacteria | 1 |  |  |
|  | Soil | Actinobacteria | 9 |  |  |
|  |  | Bacteroidetes | 2 |  |  |
|  |  | Candidatus Dadabacteria | 1 |  |  |
|  |  | Proteobacteria | 219 |  |  |
|  |  |  |  | 1 | Desert soil |

**Supplementary Table 2** | Bacteria class II PhaC genotype environmental distribution.

| Environment | Type | Phylum | Found in extreme conditions? | | |
| --- | --- | --- | --- | --- | --- |
|  |  |  | No | Yes | Extreme subtype or habitat descriptors |
| Aquatic | Freshwater | Actinobacteria | 1 |  |  |
|  |  | Proteobacteria | 20 |  |  |
|  | Marine | Actinobacteria |  | 1 | Hydrothermal vents |
|  |  | Proteobacteria | 9 |  |  |
|  |  |  |  | 1 | Deep oceanic, basalt-hosted subsurface hydrothermal fluid |
|  | None-marine saline and alkaline | Proteobacteria |  | 2 |  |
|  | Sediment | Proteobacteria | 1 |  |  |
|  | Thermal springs | Proteobacteria |  | 1 |  |
| Terrestrial | Agricultural field | Proteobacteria | 3 |  |  |
|  | Geologic | Actinobacteria | 3 |  |  |
|  |  | Proteobacteria | 1 |  |  |
|  | Mud volcano | Actinobacteria |  | 1 |  |
|  | Peat | Proteobacteria | 3 |  |  |
|  | Soil | Actinobacteria | 34 |  |  |
|  |  | Proteobacteria | 33 |  |  |

**Supplementary Table 3** | Bacteria class III PhaC genotype environmental distribution.

| Environment | Type | Phylum | Found in extreme conditions? | | | |
| --- | --- | --- | --- | --- | --- | --- |
|  |  |  | No | Yes | Extreme subtype or habitat descriptors |  |
| Aquatic | Aquaculture | Bacteroidetes | 1 |  |  |  |
|  | Freshwater | Acidobacteria | 2 |  |  |  |
|  |  | Actinobacteria | 3 |  |  |  |
|  |  | Bacteroidetes | 4 |  |  |  |
|  |  | Candidatus Blackallbacteria | 3 |  |  |  |
|  |  | Candidatus Falkowbacteria | 2 |  |  |  |
|  |  | Candidatus Kaiserbacteria | 2 |  |  |  |
|  |  | Candidatus Melainabacteria | 1 |  |  |  |
|  |  | Candidatus Moranbacteria | 1 |  |  |  |
|  |  | Candidatus Riflebacteria | 1 |  |  |  |
|  |  | Candidatus Rokubacteria | 8 |  |  |  |
|  |  | Candidatus Wallbacteria | 1 |  |  |  |
|  |  | Cyanobacteria | 2 |  |  |  |
|  |  | Elusimicrobia | 2 |  |  |  |
|  |  | Firmicutes | 5 |  |  |  |
|  |  | Nitrospirae | 1 |  |  |  |
|  |  | Proteobacteria | 51 |  |  |  |
|  |  | Spirochaetes | 2 |  |  |  |
|  | Marine | Actinobacteria | 16 |  |  |  |
|  |  |  |  | 1 | Creosote-contaminated soil |  |
|  |  |  |  | 1 | Hydrothermal vents |  |
|  |  | Bacteroidetes | 1 |  |  |  |
|  |  | Chloroflexi | 2 |  |  |  |
|  |  | Firmicutes | 2 |  |  |  |
|  |  |  |  | 1 | Hydrothermal vents |  |
|  |  | Planctomycetes 1 | 1 |  |  |  |
|  |  | Proteobacteria | 34 |  |  |  |
|  |  | Unclassified |  | 2 | Hydrothermal vents |  |
|  | Non-marine saline and alkaline | Actinobacteria |  | 2 |  |  |
|  |  | Chloroflexi |  | 1 |  |  |
|  |  | Firmicutes |  | 1 |  |  |
|  |  | Proteobacteria |  | 11 |  |  |
|  | Sediment | Actinobacteria | 2 |  |  |  |
|  |  | Bacteroidetes | 1 |  |  |  |
|  |  | Firmicutes | 1 |  |  |  |
|  |  | Proteobacteria | 1 |  |  |  |
|  | Thermal springs | Actinobacteria |  | 1 |  |  |
|  |  | Bacteroidetes |  | 2 |  |  |
|  |  | Cyanobacteria |  | 4 |  |  |
|  |  | Firmicutes |  | 1 |  |  |
|  |  | Proteobacteria |  | 3 |  |  |
| Terrestrial | Agricultural field | Proteobacteria | 2 |  |  |  |
|  | Cave | Actinobacteria | 1 |  |  |  |
|  | Geologic | Proteobacteria | 2 |  |  |  |
|  | Peat | Actinobacteria | 2 |  |  |  |
|  | Soil | Acidobacteria | 3 |  |  |  |
|  |  | Actinobacteria | 81 |  |  |  |
|  |  |  |  | 1 | Creosote-contaminated soil |  |
|  |  | candidate division NC10 | 1 |  |  |  |
|  |  | Candidatus Dadabacteria | 1 |  |  |  |
|  |  | Candidatus Falkowbacteria | 1 |  |  |  |
|  |  | Candidatus Melainabacteria | 2 |  |  |  |
|  |  | Candidatus Rokubacteria | 7 |  |  |  |
|  |  | Chloroflexi | 4 |  |  |  |
|  |  | Cyanobacteria | 2 |  |  |  |
|  |  |  |  | 1 | Desert soil |  |
|  |  | Firmicutes | 8 |  |  |  |
|  |  |  |  | 1 | Permafrost sediment |  |
|  |  | Nitrospirae | 2 |  |  |  |
|  |  | Proteobacteria | 51 |  |  |  |
|  |  | Spirochaetes | 1 |  |  |  |
|  |  | Verrucomicrobia | 1 |  |  |  |

**Supplementary Table 4** | Archaea class III PhaC genotype environmental distribution.

| Environment | Type | Phylum | Found in extreme conditions? | | | |
| --- | --- | --- | --- | --- | --- | --- |
|  |  |  | No | Yes | Extreme subtype or habitat descriptors |  |
| Aquatic | Freshwater | Euryarchaeota | 12 |  |  |  |
|  |  | Thaumarchaeota | 7 |  |  |  |
|  | Marine | Crenarchaeota | 9 |  |  |  |
|  |  | Euryarchaeota | 8 |  |  |  |
|  |  |  |  | 2 | Deep oceanic, basalt-hosted subsurface hydrothermal fluid |  |
|  |  | Thaumarchaeota | 37 |  |  |  |
|  |  |  |  | 3 | Deep sea |  |
|  |  | Unclassified |  | 1 | Hydrothermal vents |  |
|  | Non-marine saline and alkaline | Euryarchaeota |  | 42 |  |  |
|  |  | Unclassified |  | 2 |  |  |
|  | Sediment | Euryarchaeota | 3 |  |  |  |
|  | Thermal springs | Euryarchaeota |  | 3 |  |  |
|  |  | Thaumarchaeota |  | 4 |  |  |
| Terrestrial | Geologic | Euryarchaeota | 4 |  |  |  |
|  |  |  |  | 3 | Saline |  |
|  | Rock-dwelling (endoliths) | Euryarchaeota | 1 |  |  |  |
|  | Rock-dwelling (subaerial biofilms) | Euryarchaeota |  | 55 | Halite pinnacle |  |
|  | Soil | Euryarchaeota | 6 |  |  |  |
|  |  |  |  | 3 | Saline soil |  |
|  |  | Thaumarchaeota | 1 |  |  |  |
